# Race and Ethnicity and Diffusion of Telemedicine in Medicaid for Schizophrenia Care After Onset of the COVID-19 Pandemic

**DOI:** 10.1001/jamanetworkopen.2024.54776

**Published:** 2025-01-16

**Authors:** Sharon-Lise Normand, Emily Leckman-Westin, Molly Finnerty, Junghye Jeong, Jeannette Tsuei, Katya Zelevinsky, Qingxian Chen, Marcela Horvitz-Lennon

**Affiliations:** 1 Department of Health Care Policy, Harvard Medical School, Boston, Massachusetts; 2 Department of Biostatistics, Harvard T.H. Chan School of Public Health, Boston, Massachusetts; 3 New York State Office of Mental Health, Office of Population Health and Evaluation, Albany; 4 Division of Epidemiology and Biostatistics, School of Public Health, State University of New York at Albany, Rensselaer; 5 Department of Child and Adolescent Psychiatry, Grossman School of Medicine, New York University, New York; 6 Pardee RAND Graduate School, Santa Monica, California; 7 RAND, Boston, Massachusetts; 8 Department of Psychiatry, Cambridge Health Alliance and Harvard Medical School, Boston, Massachusetts

## Abstract

**Importance:**

Delivery of mental health care through telehealth (telemental health care) increased after the onset of the COVID-19 pandemic. Little is known about the speed of adoption (diffusion) of telemental health in the care in the care of individuals with schizophrenia.

**Objectives:**

To characterize telemental health care diffusion in mental health agencies serving Medicaid beneficiaries with schizophrenia and the beneficiary-level association of telemental health care use with race and ethnicity.

**Design, Setting, and Participants:**

This retrospective cohort study used New York State Medicaid data from March 1, 2019, to February 29, 2020 (prepandemic period), and from March 11, 2020, to March 31, 2021 (pandemic period), from 261 agencies serving 30 990 beneficiaries with schizophrenia with 1 or more mental health visits during the pandemic period. Statistical analysis was performed from November 2021 through September 2024.

**Exposure:**

Agency percentage of patients belonging to racial and ethnic minority groups among all Medicaid-covered patients between March 2019 and February 2020; agency type, categorized as freestanding, hospital affiliated, or state operated; beneficiary-level race and ethnicity, categorized as Asian or other (American Indian or Alaska Native; Native Hawaiian or Other Pacific Islander), Black, Latinx, White, and unknown; and pandemic severity, operationalized as COVID-19 hospitalization rates per 10 000 population in administratively defined catchment areas.

**Main Outcomes and Measures:**

Days to 10% cumulative telemental health care use within agencies, as well as beneficiary-level time to first telemental health care visit and any telemental health care visit in catchment areas in times of varying pandemic severity.

**Results:**

In this cohort study of 261 agencies (18 [7%] state operated, 79 [30%] hospital affiliated, and 164 [63%] free standing) and 30 990 beneficiaries with schizophrenia (mean [SD] age, 43 [13] years; 59% male; 7% Asian or other, 38% Black, 20% Latinx, and 25% White), 6 agencies (2%) never adopted telemental health care, and 248 (95%) reached 10% cumulative telemental health care visits in a mean of 18 days. Mean (SD) agency prepandemic shares of beneficiaries belonging to racial or ethnic minority groups (56% [23%]) were not associated with telemental health care diffusion. Diffusion was faster in state-operated vs free-standing agencies (hazard ratio [HR], 2.44 [95% CI, 1.21-4.95]). Relative to White beneficiaries, time to first telemental health care visit was slower in every racial and ethnic minority group (Asian or other: HR, 0.93 [95% CI, 0.88-0.98]; Black: HR, 0.90 [95% CI, 0.87-0.93]; Latinx: HR, 0.95 [95% CI, 0.91-0.99]). Beneficiaries from at least 1 racial or ethnic minority group were less likely than White beneficiaries to have a telemental health care visit regardless of pandemic severity and area; differences narrowed when pandemic severity was higher (eg, in New York City, the odds ratio of Black beneficiaries having a telemental health care visit relative to White beneficiaries when the pandemic severity was high was 0.70 [95% CI, 0.63-0.79] but decreased to 0.59 [95% CI, 0.53-0.67] when the pandemic severity was low).

**Conclusions and Relevance:**

In this cohort study of Medicaid beneficiaries with schizophrenia, telemental health care diffused rapidly after the onset of the COVID-19 pandemic, particularly in state-operated agencies. Together, agency-level and beneficiary-level race and ethnicity findings suggest within-agency racial and ethnic differences in diffusion of telemental health care. States should monitor the diffusion of innovations across vulnerable populations.

## Key Points

**Question:**

How rapidly did telemental health care diffuse across New York State agencies serving Medicaid beneficiaries with schizophrenia after the onset of the COVID-19 pandemic through March 31, 2021?

**Findings:**

This cohort study of 261 agencies and 30 990 beneficiaries with schizophrenia found that diffusion of telemental health care was associated with agency type but not with the racial and ethnic composition of the agencies’ patient populations. However, among beneficiaries, time to first telemental health care visit during the pandemic was slower in every racial and ethnic minority group relative to White beneficiaries.

**Meaning:**

The findings suggest that states should monitor the diffusion of innovations across vulnerable populations, particularly during emergencies.

## Teaser

This cohort study assesses the use of telemedicine in mental health agencies serving Medicaid beneficiaries with schizophrenia after the onset of the COVID-19 pandemic and examines the beneficiary-level association of telemental health care use with race and ethnicity.

## Introduction

In the US, Medicaid plays a crucial role in financing the health care received by individuals with schizophrenia,^1^ a seriously ill population with a high unmet need for pharmacologic and psychosocial mental health care that is largely delivered by specialists.^2,3^ Moreover, Medicaid beneficiaries with schizophrenia belonging to racial and ethnic minority groups, disproportionately represented in this population,^4,5^ experience racial and ethnic mental health care disparities,^6,7,8^ with the size of the disparities varying across regions.^9^ Evidence that members of racial and ethnic minority groups were slower to be treated with recently marketed second-generation antipsychotic drugs at a time when they were considered potentially more effective than older drugs^10,11,12^ suggests potential inequities in the adoption speed or diffusion of health care innovations in schizophrenia care. This finding is consistent with research suggesting that diffusion is associated with the characteristics of the innovation and with clinician, provider organizations, system-level, and patient factors including race and ethnicity.^13,14,15,16,17,18,19,20,21^

Prior to the onset of the COVID-19 pandemic, telehealth was nominally used within Medicaid due to policy and resource constraints,^22^ but flexibilities or new telehealth policies enacted after the onset of the pandemic facilitated its use to deliver care to Medicaid beneficiaries.^23,24^ Because timely access to outpatient mental health care is essential for high-need populations, including those with schizophrenia,^25^ the rapid pace of adoption of telehealth to provide care to these populations was an important goal for policymakers and provider organizations in the hardest hit areas, including New York State,^26^ the epicenter of the US COVID-19 outbreak.^27^

Studies using different methods and study cohorts have examined trends in the pandemic-era use of telehealth to deliver outpatient mental health care (henceforth, *telemental health care*) to individuals with mental illness.^28,29,30,31^ The pace at which telemental health care for individuals with schizophrenia, including those covered by Medicaid, was adopted across provider organizations during the COVID-19 pandemic and whether organizational features were associated with this diffusion are unknown. This is an important research gap—understanding whether diffusion of health care innovations varies by race and ethnicity and other factors unrelated to patients’ clinical need is vital as an issue of fairness and because unwarranted delays in access to innovations have significant outcome implications.

Although pandemic-era differences by race and ethnicity in telehealth use have been studied,^32,33,34,35,36,37,38^ few studies have focused on telemental health care use among publicly insured adults treated by different provider organizations in different regions.^29,39^ We are unaware of research examining if the stress experienced by the health care system due to unexpected shocks modifies the association of race and ethnicity with access to telemental health care, or if race and ethnicity are associated with time to telemental health care use, another indicator of access to the innovation. Therefore, we characterized the diffusion of telemental health care in New York State’s outpatient mental health agencies serving Medicaid beneficiaries with schizophrenia during the year that followed the onset of the pandemic and examined whether agency shares of beneficiaries from racial and ethnic minority groups and other agency characteristics were associated with adoption speed. We also assessed if race and ethnicity were associated with time to telemental health care use and how this association varied by geographic area and health system stress levels.

## Methods

The institutional review boards at Harvard Medical School, RAND, and the Nathan Kline Institute for Psychiatric Research reviewed and approved this cohort study. Because we used secondary data that were deidentified for the purposes of conducting this research under institutional review board approval granted by the Nathan Kline Institute for Psychiatric Research, individual consent was not required. The Strengthening the Reporting of Observational Studies in Epidemiology (STROBE) reporting guideline was followed.

### Data Collection and Study Population

We used New York State Medicaid data for the periods covering March 1, 2019, to February 29, 2020 (prepandemic period), and March 11, 2020, to March 31, 2021 (pandemic period). March 11, 2020, was selected as the start of the pandemic period because that is the date when the state updated telehealth emergency regulations to facilitate care delivery via telehealth, a regulatory decision that removed a critical barrier to telehealth adoption. Our data source was the New York Medicaid Data Warehouse maintained by the Office of Mental Health (OMH), the state’s mental health Medicaid authority. Data extracted included beneficiary characteristics (enrollment, date of birth, race and ethnicity [Asian or other (American Indian or Alaska Native; and Native Hawaiian or Other Pacific Islander), Black, Latinx, White, and unknown], and Supplemental Security Income [SSI] as a mechanism for Medicaid eligibility) and services rendered (dates of services, diagnoses, procedures, and state-licensed outpatient mental health provider agencies [henceforth, *agencies*]) and whether care was delivered in person or via telehealth. Medicaid agencies collect self-reported race and ethnicity information through a paper or online application during the eligibility determination and enrollment processes, with additional data collected during the renewal or redetermination processes in some states, including New York State. We focused on beneficiaries aged 18 to 64 years having (1) at least 1 month of Medicaid enrollment during the pandemic period, (2) a primary or secondary diagnosis of schizophrenia as indicated by at least 1 inpatient or outpatient claim with *International Statistical Classification of Diseases and Related Health Problems, Tenth Revision* (*ICD-10*) codes for schizophrenia (F20, F25), and (3) at least 1 mental health visit during the pandemic period regardless of the primary diagnosis for the encounter. Dual Medicare or Medicaid beneficiaries were excluded due to incomplete information on Medicare-covered services. We required only 1 month of enrollment because some individuals could become newly eligible during the pandemic. Continuous enrollment restrictions would restrict the cohort, therefore limiting the understanding of the diffusion patterns of a new care-enabling technology. Although there are no strict guidelines on the optimal frequency of outpatient visits, individuals with schizophrenia with acute presentations requiring close symptom or treatment response may need to be seen frequently, every 2 to 4 weeks, while individuals with stable presentations may be seen every 2 to 4 months.

### Measuring Telemental Health Care Visits

We identified telemental health care visits using telehealth procedure modifiers (GT, 95 [modifier codes used to indicate that a code described by Healthcare Common Procedure Coding System (HCPCS) procedure code was delivered via telehealth]). In New York State, telemental health care standards were first established in 2015. Although the OMH expanded regulations in July 2019 to allow a broader range of agencies to deliver mental health care through telehealth, telemental health care use prior to the pandemic was negligible, less than 1% statewide.^29^

### Primary Outcome: Diffusion of Telemental Health Care in Agencies

We described the cumulative percentage of telemental health care adopters over time, with a focus on agencies as the unit of interest. For each agency, we assessed the number of days to various targets. The targets were defined as the time from March 11, 2020, to the day in which telemental health care was used in a specific percentage of the cumulative mental health outpatient visits of the Medicaid beneficiaries with schizophrenia in the agency (eAppendix 1 in Supplement 1). The targets ranged by 10–percentage point increments from 10%, which characterizes routine use,^40,41,42^ to 90%. By construction, agencies meeting a higher target also meet the lower target. Our primary outcome was the 10% target.

### Agency Characteristics

State-licensed mental health agencies were identified using state-specific rate codes.^43^ We characterized agencies using information extracted from all visits for all Medicaid patients, regardless of diagnosis, occurring during the prepandemic period (March 1, 2019, to February 29, 2020). The mean (SD) daily number of mental health outpatient visits and the percentages (SDs) of beneficiaries belonging to racial and ethnic minority groups, having SSI (a disability indicator), and having a schizophrenia diagnosis were identified. Because beneficiaries with substance use disorders (SUDs) represent another vulnerable population whose high need for timely care is often unmet,^44,45^ we also examined agency shares of beneficiaries having an opioid use disorder (OUD) diagnosis or other SUD diagnosis (nonopioid SUD). Beneficiary diagnosis (schizophrenia, OUD, or nonopioid SUD) was based on observation of at least 1 inpatient or outpatient claim with the relevant *ICD-10* codes as primary or secondary diagnoses. Agencies were characterized based on their ownership and degree of regulatory oversight by the New York State Department of Health, the state Medicaid authority, and the OMH and grouped into 3 categories. Free-standing agencies are state-regulated agencies having no hospital affiliation, hospital-affiliated agencies are state-regulated agencies that are owned and operated by hospitals, and state-operated agencies are owned, operated, and regulated by the OMH. Agencies’ geographic locations were defined based on the OMH’s 5 administrative catchment areas: New York City (NYC), Long Island, Hudson River, Central, and Western.

### Secondary Outcomes: Beneficiary Telemental Health Care Use

We defined 2 secondary outcomes at the beneficiary level, both measures of access to telemental health care: time to first telemental health care visit, measured as number of days from March 11, 2020, and a binary outcome indicating any telemental health care visit during the pandemic period.

### Beneficiary Characteristics

Because the beneficiary-level models required only 1 month of Medicaid enrollment as of the date when telehealth regulations were updated after the pandemic onset (March 11, 2020), beneficiary characteristics were obtained using information captured during the period between March 11, 2020, through the first mental health outpatient visit (whether in person or telehealth), inclusive of both dates. We determined race and ethnicity, sex, age, county of residence, and the following illness severity measures: receipt of SSI and 3 SUD-related measures (OUD and nonopioid SUD comorbidity, and participation in an opioid treatment program, which entails receipt of buprenorphine or methadone).

### Statistical Analysis

#### Primary Analysis: Measuring Agency-Level Diffusion Outcomes

Statistical analysis was performed from November 2021 through September 2024. We plotted cumulative days to 10%, 50%, and 90% targets for agencies and examined the racial and ethnic distributions of beneficiaries served by agencies meeting each target at the time of meeting the target and for agencies never adopting telemental health care for schizophrenia, using agency characteristics measured during the prepandemic period. A Cox proportional hazards regression model estimated time to the 10% target and hazard ratios (HRs) with 95% CIs for an increase of 1 SD for each agency characteristic; if the agency did not reach the 10% target during the pandemic period, the observation was censored. We repeated this model using the 50% target to assess the consistency of the regression coefficients.

#### Secondary Analysis: Measuring Beneficiary Access to Telemental Health Care and Its Association With Race and Ethnicity

We estimated Cox proportional hazards regression models to examine the association of beneficiary race and ethnicity and time to first telemental health care visit, censoring beneficiaries not having telemental health care visits. Beneficiary characteristics included sex, age, county of residence, and the 4 severity measures. Hazard ratios and 95% CIs were estimated.

#### Measuring Beneficiary Telemental Health Care Access, Association With Race and Ethnicity, and Modification of the Association by Health Care System Stress

We selected 2 of the state’s 5 administrative catchment areas that differed by level of health care system stress (operationalized using COVID-19 hospitalization rates) and racial and ethnic composition. We categorized COVID-19 hospitalization rates per 10 000 overall population (ie, all area residents regardless of age and payer). The COVID-19 pandemic affected NYC and Long Island earliest, with the Central and Western catchment areas (henceforth *Central* or *Western region*) having the lowest COVID-19 hospitalization rates (eFigure 1 in Supplement 1). COVID-19 hospitalization rates decreased across all catchment areas between May and October 2020 and then increased equally across all catchment areas, delineating 3 distinct periods (period 1: March 11 to April 30, 2020; period 2: July 9 to August 20, 2020; period 3: December 24, 2020, to February 4, 2021). Individuals belonging to racial or ethnic minority groups comprised 78% of NYC beneficiaries vs less than half in the Central or Western region in each of the 3 time periods, with Black beneficiaries representing the largest minority group.

Using NYC and the Central or Western regions only, we modeled the log odds of the probability of having at least 1 telemental health care visit among beneficiaries with schizophrenia having any mental health care use. Beneficiary-level covariates included race and ethnicity, receipt of SSI, OUD and nonopioid SUD comorbidity, participation in an opioid treatment program, time period, and catchment area; the pairwise interaction of race and ethnicity, time period, and catchment area; and their 3-way interaction (eAppendix 2 in Supplement 1). The interactions quantified how stress to the health care system, measured by COVID-19 hospitalization rates, modified the associations of telemental health care access with the patient’s race and ethnicity, adjusting for the catchment area’s racial and ethnic composition. We computed the odds ratios and 95% CIs of telemental health care visits for individuals belonging to racial and ethnic minority groups vs those of White race within catchment area time periods and across time periods.

We made no adjustment for multiplicity of testing, so we report no *P* values. Analyses were conducted using the SAS Enterprise Guide, version 8.2.0.1201 (64-bit) (SAS Institute Inc).

## Results

In the prepandemic period (March 1, 2019, to February 29, 2020), we identified 261 agencies having at least 1 mental health clinic visit: 164 (63%) were free standing, 79 (30%) were hospital affiliated, and 18 (7%) were state operated (eFigure 2 in Supplement 1). The agency mean (SD) daily number of mental health outpatient visits regardless of beneficiary diagnosis was 54 (72), the mean (SD) percentage of beneficiaries belonging to racial or ethnic minority groups was 56% (23%), and the mean (SD) percentage of beneficiaries with schizophrenia was 20% (17%) (Table 1).

**Table 1.** Characteristics of New York State Mental Health Agencies Assessed With Respect to Their Medicaid Beneficiary Populations, March 1, 2019, to February 29, 2020

Characteristic	Value (N = 261)
Daily mental health outpatient visits, mean (SD), No.	54 (72)
Beneficiaries belonging to racial or ethnic minority groups, mean (SD), %	56 (23)
Beneficiaries with Supplemental Security Income, mean (SD), %	42 (16)
Beneficiaries having opioid use disorder, mean (SD), %	11 (10)
Beneficiaries having opioid use disorder and participating in an opioid treatment program, mean (SD), %	3 (6)
Beneficiaries having nonopioid substance use disorder, mean (SD), %	42 (17)
Beneficiaries having schizophrenia, mean (SD), %	20 (17)
Agency type, No. (%)	
Free standing	164 (63)
Hospital affiliated	79 (30)
State operated	18 (7)

During the pandemic period (March 11, 2020, to March 31, 2021), the 261 agencies provided care to 30 990 beneficiaries with schizophrenia (mean [SD] age, 43 [13] years; 59% male and 41% female; 7% Asian or other, 38% Black, 20% Latinx, and 25% White; 61% SSI recipients) who made a total of 763 474 mental health outpatient visits (Table 2). A total of 7% of beneficiaries had a comorbid OUD, but only 3% participated in an opioid treatment program, and 26% had nonopioid SUD. The mean (SD) daily number of mental health outpatient visits ranged from 131 (77) for beneficiaries of Asian or other race to 731 (429) for Black beneficiaries, with the share of telemental health care to in-person visits smallest for Black beneficiaries (1.86) and largest for White beneficiaries (2.18) (eFigure 3 in Supplement 1).

**Table 2.** Characteristics of Adult Medicaid Beneficiaries With Schizophrenia Treated at 261 New York State Mental Health Agencies From March 11, 2020, to March 31, 2021, at Time of First Mental Health Outpatient Visit

Characteristic	No. (%) (N = 30 990)
Male	18 178 (59)
Female	12 812 (41)
Aged 51-64 y	10 567 (34)
Race and ethnicity	
Asian or other^a^	1999 (7)
Black	11 786 (38)
Latinx	6289 (20)
White	7818 (25)
Unknown	3098 (10)
Supplemental Security Income recipient	18 850 (61)
Opioid use disorder comorbidity	2085 (7)
Participating in an opioid treatment program	920 (3)
Nonopioid substance use disorder comorbidity	8105 (26)
Agency type, No. (%)	
Free standing	13 239 (43)
Hospital affiliated	14 145 (46)
State operated	3606 (12)

^a^ Other includes American Indian or Alaska Native and Native Hawaiian or Other Pacific Islander.

### Agency Diffusion Outcomes

**Figure 1.**
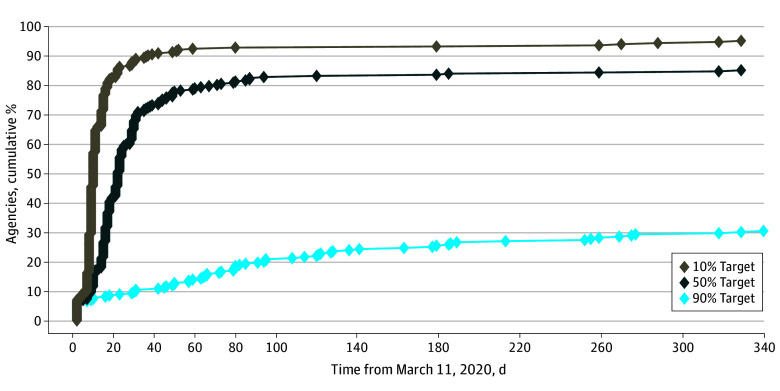
Diffusion of Telemental Health Care for Schizophrenia Cumulative percentage of Medicaid agencies using telemental health care by day from March 11, 2020, for 3 different targets.

Across agencies, the mean (SD) number of mental health outpatient visits per beneficiary with schizophrenia during the pandemic period was 25 (20) and did not vary by race and ethnicity. Diffusion was rapid, with 248 agencies (95%) reaching the 10% target in a mean (SD) of 18 (42) days, 222 (85%) reaching the 50% target in 28 (40) days, and 83 (32%) reaching the 90% target in 97 (102) days (Figure 1; eFigure 4 in Supplement 1). Six agencies (2%) never adopted telemental health care for schizophrenia and treated mostly White beneficiaries with schizophrenia (3% Black, 3% Latinx, and 73% White), for a total of 2641 in-person–only mental health outpatient visits.

Thirteen agencies (5%) did not reach the 10% target. Agencies with higher mean daily mental health visits during the prepandemic period were quicker to reach the 10% target (HR, 1.14 [95% CI, 1.01-1.29]; Table 3). Agency shares of beneficiaries belonging to racial and ethnic minority groups, beneficiaries with SSI, beneficiaries with OUD regardless of opioid treatment program participation, and beneficiaries with nonopioid SUD during the prepandemic period were not associated with speed of reaching the 10% target; however, a larger prepandemic share of beneficiaries with schizophrenia was associated with faster speed of reaching the 10% target (HR, 1.53 [95% CI, 1.26-1.87] per 1-SD increase). Compared with free-standing agencies, telemental health care diffused faster across state-operated mental health agencies (HR, 2.44 [95% CI, 1.21-4.95]) and slower across hospital-affiliated agencies (HR, 0.37 [95% CI, 0.27-0.52]). Associations were generally consistent for the 50% target.

**Table 3.** Hazard Ratios of the Number of Days to Reach 10% and 50% Cumulative Telemental Health Care Visits in 261 New York State Mental Health Agencies

Covariate	Increase in covariate	Hazard ratio (95% CI)^a^	
		10% Target	50% Target
Mean daily mental health outpatient visits, No.	72	1.14 (1.01-1.29)	1.05 (0.92-1.19)
Minoritized beneficiaries, %	23	0.90 (0.77-1.05)	0.93 (0.79-1.09)
Beneficiaries with Supplemental Security Income, %	16	1.05 (0.87-1.26)	0.96 (0.80-1.16)
Beneficiaries with opioid use disorder, %	10	0.81 (0.58-1.12)	0.76 (0.52-1.11)
Beneficiaries with opioid use disorder and participating in an opioid treatment program, %	6	1.08 (0.85-1.38)	1.08 (0.81-1.46)
Beneficiaries with nonopioid substance use disorder, %	17	0.94 (0.76-1.17)	0.88 (0.69-1.11)
Beneficiaries with schizophrenia, %	17	1.53 (1.26-1.87)	1.39 (1.12-1.74)
Agency type			
Free standing	1 [Reference]	1 [Reference]	1 [Reference]
Hospital affiliated	NA	0.37 (0.27-0.52)	0.27 (0.19-0.39)
State operated	NA	2.44 (1.21-4.95)	3.38 (1.66-6.89)

Abbreviation: NA, not applicable.

^a^ Hazards computed for a 1-SD increase in the covariate except for agency type. A total of 13 of 261 agencies (5%) were censored at the 10% target, and 39 of 261 agencies (15%) were censored at the 50% target.

### Beneficiary Access to Telemental Health Care and Its Association With Race and Ethnicity

At the beneficiary level, time to first telemental health care visit was slower in every racial or ethnic minority group relative to White beneficiaries (Asian or other: HR, 0.93 [95% CI, 0.88-0.98]; Black: HR, 0.90 [95% CI, 0.87-0.93]; Latinx: HR, 0.95 [95% CI, 0.91-0.99]) (eTable in Supplement 1). Time to first telemental health care visit was also slower for non-SSI recipients relative to SSI recipients (HR, 0.94 [95% CI, 0.91-0.96]), those with OUD (HR, 0.68 [95% CI, 0.63-0.73]), and those with nonopioid SUD (HR, 0.55 [95% CI, 0.53-0.56]). Access was faster among female beneficiaries (HR, 1.07 [95% CI, 1.04-1.10]) relative to male beneficiaries and among beneficiaries with OUD who participated in an OUD treatment program relative to those not participating (HR, 1.41 [95% CI, 1.27-1.56]). Fifteen percent (n = 4542) of the beneficiaries were censored.

### Beneficiary Telemental Health Care Access, Association With Race and Ethnicity, and Modification of the Association by Health Care System Stress

**Figure 2.**
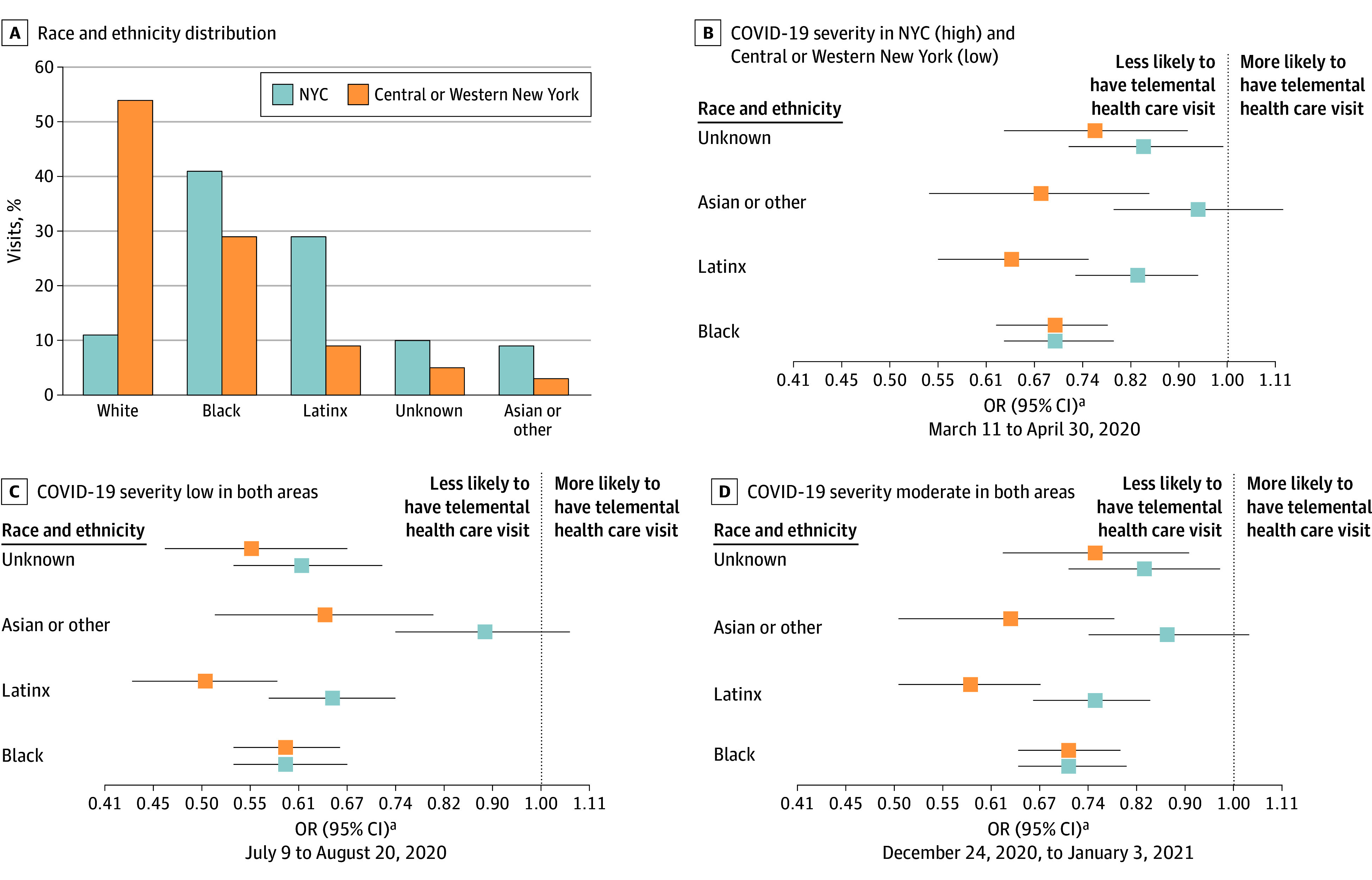
Beneficiary Telemental Health Care Access as a Function of Race and Ethnicity, Health Care System Stress, and Catchment Area A, Race and ethnicity distribution by catchment area. B, With COVID-19 severity high in New York City (NYC) and low in Central or Western New York, the odds of at least 1 telemental health care visit among those having mental health outpatient visits are stratified by time period. C, With COVID-19 severity low in both areas, odds of at least 1 telemental health care visit among those having mental health outpatient visits, stratified by time period. D, With COVID-19 severity moderate in both areas, the odds of at least 1 telemental health care visit among those having mental health outpatient visits are stratified by time period. Odds ratios (ORs) are adjusted for age, sex, and severity variables (Supplemental Security Income, opioid use disorder, and nonopioid substance abuse disorder comorbidity, and participation in an opioid treatment program). ^a^Reference: White participants.

Relative to White beneficiaries and regardless of COVID-19 severity period, beneficiaries belonging to racial and ethnic minority groups in the Central or Western region were less likely to have a telemental health care visit, while in NYC, this was true for all beneficiaries except Asian or other beneficiaries. In both catchment areas, when COVID-19 severity was low (July 9 to August 20, 2020), the differences between White beneficiaries and both Black and Latinx beneficiaries were larger than when COVID-19 severity was high (Figure 2).

## Discussion

Our study shows that after the onset of the COVID-19 pandemic, telemental health care diffused rapidly to deliver care to Medicaid beneficiaries with schizophrenia across New York’s state-licensed mental health agencies. This is evidenced by most agencies having reached the diffusion target indicating routine use in less than 3 weeks, which may be an adequate spacing of visits for individuals with acute presentations of schizophrenia. This is an unsurprising finding given the severity of the public health emergency. Although agencies with larger prepandemic volumes of patients with schizophrenia adopted telemental health care faster, the agency prepandemic racial and ethnic distribution of beneficiaries was not associated with diffusion of telemental health care. However, for beneficiaries with schizophrenia, telemental health care access measured through time to first telemental health care visit was slower for beneficiaries from racial and ethnic minority groups relative to White beneficiaries. These findings suggest within-agency racial and ethnic differences in adoption speed.

Because little is known about racial and ethnic differences in the diffusion of telemental health care within the Medicaid program, we are unable to ground our findings in the larger literature. However, observed differences among beneficiaries with schizophrenia align with evidence of disparities in diffusion of novel antipsychotic drugs^11^ and telephone and video-enabling technology access inequities.^46^ When focusing on telemental health care access across geographic regions having different racial and ethnic compositions and varying levels of health care system stress, we also observed race and ethnicity differences. The observed differences were somewhat attenuated when the severity of schizophrenia was greater, suggesting that vulnerable groups may have been prioritized when the health care system was under more stress.

The finding that state-operated agencies delivering care to Medicaid beneficiaries with schizophrenia were faster to adopt telemental health care relative to the other agency types may stem from their greater familiarity with state government procedures, permitting rapid implementation of new state policies and regulations. This result is consistent with evidence that access to financial and other resources is a key factor in the diffusion of health care technologies.^47,48,49,50,51,52^ The finding that hospital-affiliated agencies were slower to adopt telemental health care compared with free-standing agencies may be associated with the free-standing agencies’ greater nimbleness, as they tend to be smaller and more vertically integrated and have a more cohesive workforce that is exclusively focused on mental health care. Although these features may not be easy to replicate across all mental health care provider organizations, greater administrative and workforce integration is a desirable feature for any organization.^53,54^

### Limitations

Our study has some limitations. First, our beneficiary-level analyses adjusted for a partial set of beneficiary-level severity indicators—consequently, we cannot rule out that HRs resulting from the analysis of time to first telemental health care visit may reflect need differences. The trade-off is that adjusting for other severity indicators, such as prior service use, required longer continuous enrollment preceding the first outpatient visit, thus precluding the assessment of the state’s health care system’s emergency response capacity. We also focused on those having at least 1 mental health visit in the postpandemic period. Second, our study may not be generalizable to other states or payers. Third, lack of information in the New York Medicaid data on whether telehealth was used through audiovisual vs audio-only services is a limitation given evidence of racial and ethnic differences in the use of these services.^37^

## Conclusions

In this cohort study of adults with Medicaid in New York State, telemental health care for schizophrenia care diffused rapidly after onset of the COVID-19 pandemic and was faster in state-operated agencies. Agency-level and beneficiary-level race and ethnicity findings suggest within-agency racial and ethnic differences in diffusion of telemental health care. More research is needed to confirm and expand our novel diffusion findings, but some steps may be taken immediately. States should monitor the diffusion of innovations among vulnerable populations, investigating whether adoption speed varies by race and ethnicity. States should also examine factors associated with access to the innovation, paying particular attention to the role of agencies, and implement corrective strategies, such as financial incentives targeted to underperforming agencies if evidence of inequities is found.

## Notes

Supplement 1. **eAppendix 1.** Agency Level Diffusion Outcome: Approach and Methodological Details **eAppendix 2.** Beneficiary Likelihood of Any Telemental Health Visit, Its Association With Race and Ethnicity, and Modification of the Association by Healthcare System Stress: Details on Methods **eTable.** Hazard Ratios of Time to First Telemental Health Visit in New York State Among 30 990 Medicaid Adults With Schizophrenia **eFigure 1**. COVID-19 Hospitalization Rates (per 10 000) Reflect Varying Disease Severity, by Date and New York State Catchment Area **eFigure 2**. CONSORT Diagram **eFigure 3**. Average Daily Telemental Health Visits Relative to Average In-Person Mental Health Outpatient Visits for New York State Medicaid Beneficiaries With Schizophrenia, Across Agencies **eFigure 4**. Days to Diffusion Targets
eReferences.

Supplement 2.
Data Sharing Statement

## References

[1] Frank RG, Glied SA. Better But Not Well: Mental Health Policy in the United States Since 1950. Johns Hopkins University Press; 2006. doi:10.1353/book.3252

[2] Horvitz-Lennon M, Donohue JM, Domino ME, Normand SL. Improving quality and diffusing best practices: the case of schizophrenia. Health Aff (Millwood). 2009;28(3):701-712. doi:10.1377/hlthaff.28.3.701 19414878 PMC2832306

[3] Kotov R, Fochtmann L, Li K, . Declining clinical course of psychotic disorders over the two decades following first hospitalization: evidence from the Suffolk County Mental Health Project. Am J Psychiatry. 2017;174(11):1064-1074. doi:10.1176/appi.ajp.2017.16101191 28774193 PMC5767161

[4] Bresnahan M, Begg MD, Brown A, . Race and risk of schizophrenia in a US birth cohort: another example of health disparity? Int J Epidemiol. 2007;36(4):751-758. doi:10.1093/ije/dym041 17440031

[5] Regier DA, Robins LN. Psychiatric Disorders in America: The Epidemiologic Catchment Area Study. Maxwell Macmillan International; 1991.

[6] Horvitz-Lennon M, Volya R, Donohue JM, Lave JR, Stein BD, Normand SL. Disparities in quality of care among publicly insured adults with schizophrenia in four large U.S. states, 2002-2008. Health Serv Res. 2014;49(4):1121-1144. doi:10.1111/1475-6773.12162 24628414 PMC4111783

[7] Normand SL, Zelevinsky K, Finnerty M, . Racial-ethnic disparities in quality of care among Medicaid beneficiaries with schizophrenia. Psychiatr Serv. 2024;75(10):969-978. doi:10.1176/appi.ps.20230564 38863327 PMC12044604

[8] Normand ST, Zelevinsky K, Abing HK, Horvitz-Lennon M. Statistical approaches for quantifying the quality of neurosurgical care. World Neurosurg. 2022;161:331-342. doi:10.1016/j.wneu.2022.01.047 35505552 PMC9074098

[9] Horvitz-Lennon M, Volya R, Garfield R, Donohue JM, Lave JR, Normand SLT. Where you live matters: quality and racial/ethnic disparities in schizophrenia care in four state Medicaid programs. Health Serv Res. 2015;50(5):1710-1729. doi:10.1111/1475-6773.12296 25759240 PMC4600368

[10] Horvitz-Lennon M, Alegría M, Normand SL. The effect of race-ethnicity and geography on adoption of innovations in the treatment of schizophrenia. Psychiatr Serv. 2012;63(12):1171-1177. doi:10.1176/appi.ps.201100408 23026838 PMC3666934

[11] Opolka JL, Rascati KL, Brown CM, Gibson PJ. Ethnicity and prescription patterns for haloperidol, risperidone, and olanzapine. Psychiatr Serv. 2004;55(2):151-156. doi:10.1176/appi.ps.55.2.151 14762239

[12] Valenstein M, McCarthy JF, Ignacio RV, Dalack GW, Stavenger T, Blow FC. Patient- and facility-level factors associated with diffusion of a new antipsychotic in the VA health system. Psychiatr Serv. 2006;57(1):70-76. doi:10.1176/appi.ps.57.1.70 16399965

[13] Wisdom JP, Chor KH, Hoagwood KE, Horwitz SM. Innovation adoption: a review of theories and constructs. Adm Policy Ment Health. 2014;41(4):480-502. doi:10.1007/s10488-013-0486-4 23549911 PMC3894251

[14] Rogers EM. Diffusion of Innovations. 5th ed. Free Press; 2003.

[15] Greenhalgh T, Robert G, Macfarlane F, Bate P, Kyriakidou O. Diffusion of innovations in service organizations: systematic review and recommendations. Milbank Q. 2004;82(4):581-629. doi:10.1111/j.0887-378X.2004.00325.x 15595944 PMC2690184

[16] Drake R, Skinner J, Goldman HH. What explains the diffusion of treatments for mental illness? Am J Psychiatry. 2008;165(11):1385-1392. doi:10.1176/appi.ajp.2008.08030334 18981070 PMC2647364

[17] Backer TE, Liberman RP, Kuehnel TG. Dissemination and adoption of innovative psychosocial interventions. J Consult Clin Psychol. 1986;54(1):111-118. doi:10.1037/0022-006X.54.1.111 3958295

[18] Trajtenberg M, Yitzhaki S. The diffusion of innovations: a methodological reappraisal. J Bus Econ Stat. 1989;7(1):35-47. doi:10.1080/07350015.1989.10509711

[19] Mallinson DJ. Building a better speed trap: measuring policy adoption speed in the American states. State Polit Policy Q. 2016;16(1):98-120. doi:10.1177/1532440015596088

[20] Skinner J, Staiger D. Technology diffusion and productivity growth in health care. Rev Econ Stat. 2015;97(5):951-964. doi:10.1162/REST_a_00535 26989267 PMC4792131

[21] Groeneveld PW, Laufer SB, Garber AM. Technology diffusion, hospital variation, and racial disparities among elderly Medicare beneficiaries: 1989-2000. Med Care. 2005;43(4):320-329. doi:10.1097/01.mlr.0000156849.15166.ec 15778635

[22] Douglas MD, Xu J, Heggs A, Wrenn G, Mack DH, Rust G. Assessing telemedicine utilization by using Medicaid claims data. Psychiatr Serv. 2017;68(2):173-178. doi:10.1176/appi.ps.201500518 27691381 PMC5444290

[23] Center for Connected Health Policy. COVID-19 telehealth coverage policies. Updated March 2021. Accessed September 21, 2023. https://www.cchpca.org/resources/covid-19-telehealth-coverage-policies/

[24] Chen PG, Heins SE, Dellva S. State Medicaid telehealth coverage policy decisions since the COVID-19 public health emergency. PR-A2089-1. Office of the Assistant Secretary for Planning and Evaluation. May 2023. Accessed September 15, 2023. https://aspe.hhs.gov/sites/default/files/documents/11bc151081feb0123fc80283874ab7af/medicaid-telehealth.pdf

[25] Horvitz-Lennon M, Leckman-Westin E, Finnerty M, . Healthcare access for a diverse population with schizophrenia following the onset of the COVID-19 pandemic. Community Ment Health J. 2024;60(1):72-80. doi:10.1007/s10597-023-01105-1 37199854 PMC10193305

[26] Erlich MD, Casoy F, Berezin J, Hernandez Y, Smith TE. Building and landing the plane while flying: how New York State addressed the needs of people with serious mental illness during the COVID-19 pandemic. Schizophr Bull Open. 2022;3(1):sgac035. doi:10.1093/schizbullopen/sgac035 36348646 PMC9620771

[27] Bialek S, Bowen V, Chow N, ; CDC COVID-19 Response Team. Geographic differences in COVID-19 cases, deaths, and incidence—United States, February 12-April 7, 2020. MMWR Morb Mortal Wkly Rep. 2020;69(15):465-471. doi:10.15585/mmwr.mm6915e4 32298250 PMC7755058

[28] Zhu JM, Myers R, McConnell KJ, Levander X, Lin SC. Trends in outpatient mental health services use before and during the COVID-19 pandemic. Health Aff (Millwood). 2022;41(4):573-580. doi:10.1377/hlthaff.2021.01297 35377763 PMC9056059

[29] Bareis N, Tepper MC, Wang R, . Engagement of individuals with serious mental illness in outpatient mental health services and telehealth use during the COVID-19 pandemic. Psychiatry Res. 2023;329:115497. doi:10.1016/j.psychres.2023.115497 37778232 PMC10842636

[30] Ainslie M, Brunette MF, Capozzoli M. Treatment interruptions and telemedicine utilization in serious mental illness: retrospective longitudinal claims analysis. JMIR Ment Health. 2022;9(3):e33092. doi:10.2196/33092 35311673 PMC8981005

[31] Cantor J, Schuler MS, Matthews S, Kofner A, Breslau J, McBain RK. Availability of mental telehealth services in the US. JAMA Health Forum. 2024;5(2):e235142. doi:10.1001/jamahealthforum.2023.5142 38306092 PMC10837750

[32] Eberly LA, Kallan MJ, Julien HM, . Patient characteristics associated with telemedicine access for primary and specialty ambulatory care during the COVID-19 pandemic. JAMA Netw Open. 2020;3(12):e2031640. doi:10.1001/jamanetworkopen.2020.31640 33372974 PMC7772717

[33] Patel SY, Mehrotra A, Huskamp HA, Uscher-Pines L, Ganguli I, Barnett ML. Variation in telemedicine use and outpatient care during the COVID-19 pandemic in the United States. Health Aff (Millwood). 2021;40(2):349-358. doi:10.1377/hlthaff.2020.01786 33523745 PMC7967498

[34] Weber E, Miller SJ, Astha V, Janevic T, Benn E. Characteristics of telehealth users in NYC for COVID-related care during the coronavirus pandemic. J Am Med Inform Assoc. 2020;27(12):1949-1954. doi:10.1093/jamia/ocaa216 32866249 PMC7499577

[35] Whaley CM, Pera MF, Cantor J, . Changes in health services use among commercially insured US populations during the COVID-19 pandemic. JAMA Netw Open. 2020;3(11):e2024984. doi:10.1001/jamanetworkopen.2020.24984 33151319 PMC7645698

[36] White-Williams C, Liu X, Shang D, Santiago J. Use of telehealth among racial and ethnic minority groups in the United States before and during the COVID-19 pandemic. Public health Rep. 2023;138(1):149-156. doi:10.1177/00333549221123575 36113138 PMC9482875

[37] Karimi M, Lee EC, Couture SJ, . National survey trends in telehealth use in 2021: disparities in utilization and audio vs. video services. HP-2022-04. Office of the Assistant Secretary for Planning and Evaluation; US Department of Health and Human Services. February 1, 2022. Accessed October 30, 2024. https://aspe.hhs.gov/sites/default/files/documents/4e1853c0b4885112b2994680a58af9ed/telehealth-hps-ib.pdf

[38] Reed ME, Huang J, Graetz I, . Patient characteristics associated with choosing a telemedicine visit vs office visit with the same primary care clinicians. JAMA Netw Open. 2020;3(6):e205873. doi:10.1001/jamanetworkopen.2020.5873 32585018 PMC7301227

[39] Marcondes FO, Normand ST, Le Cook B, . Racial and ethnic differences in telemedicine use. JAMA Health Forum. 2024;5(3):e240131. doi:10.1001/jamahealthforum.2024.0131 38517424 PMC10960201

[40] Keating NL, Huskamp HA, Schrag D, . Diffusion of bevacizumab across oncology practices: an observational study. Med Care. 2018;56(1):69-77. doi:10.1097/MLR.0000000000000840 29135615 PMC5726588

[41] Gilstrap LG, Blair RA, Huskamp HA, Zelevinsky K, Normand SL. Assessment of second-generation diabetes medication initiation among Medicare enrollees from 2007 to 2015. JAMA Netw Open. 2020;3(5):e205411. doi:10.1001/jamanetworkopen.2020.5411 32442290 PMC7244990

[42] Griliches Z. Hybrid corn: an exploration in the economics of technological change. Econometrica. 1957;25(4):501-522. doi:10.2307/1905380

[43] New York State Office of Mental Health. Guidance documents. Accessed November 21, 2024. https://omh.ny.gov/omhweb/guidance

[44] Liu J, Storfer-Isser A, Mark TL, . Access to and engagement in substance use disorder treatment over time. Psychiatr Serv. 2020;71(7):722-725. doi:10.1176/appi.ps.201800461 32089081

[45] O’Brien P, Henke RM, Schaefer MB, Lin J, Creedon TB. Utilization of treatment by Medicaid enrollees with opioid use disorder and co-occurring substance use disorders. Drug Alcohol Depend. 2020;217:108261. doi:10.1016/j.drugalcdep.2020.108261 32979735

[46] Andrew P, Erica T. Smartphones help Blacks, Hispanics bridge some—but not all—digital gaps with Whites. Pew Research Center. Updated August 20, 2019. Accessed August 2, 2023. https://policycommons.net/artifacts/616650/smartphones-help-blacks-hispanics-bridge-some/

[47] Cappellaro G, Ghislandi S, Anessi-Pessina E. Diffusion of medical technology: the role of financing. Health Policy. 2011;100(1):51-59. doi:10.1016/j.healthpol.2010.10.004 21055840

[48] Donohue JM, Normand SL, Horvitz-Lennon M, Men A, Berndt ER, Huskamp HA. Regional variation in physician adoption of antipsychotics: impact on US Medicare expenditures. J Ment Health Policy Econ. 2016;19(2):69-78. 27453458 PMC5020418

[49] Freeborn DK, Baer D, Greenlick MR, Bailey JW. Determinants of medical care utilization: physicians’ use of laboratory services. Am J Public Health. 1972;62(6):846-853. doi:10.2105/AJPH.62.6.846 5032012 PMC1530333

[50] Gu C, Huskamp H, Donohue J, Normand SL. A bayesian hierarchical model for characterizing the diffusion of new antipsychotic drugs. Biometrics. 2021;77(2):649-660. doi:10.1111/biom.13324 32627176 PMC8108482

[51] Russell LB. Technology in Hospitals: Medical Advances and Their Diffusion. Brookings Institution; 1979. doi:10.1097/00004010-197904040-00011

[52] Williamson PM. The adoption of new drugs by doctors practising in group and solo practice. Soc Sci Med (1967). 1975;9(4-5):233-236. doi:10.1016/0037-7856(75)90027-x 1154044

[53] Barki H, Pinsonneault A. A model of organizational integration, implementation effort, and performance. Organ Sci. 2005;16(2):165-179. doi:10.1287/orsc.1050.0118

[54] Van de Ven AH, Rogers RW, Bechara JP, Sun K. Organizational diversity, integration and performance. J Organ Behav. 2008;29(3):335-354. doi:10.1002/job.511

