# Supplementary material for: Race and Ethnicity and Diffusion of Telemedicine in Medicaid for Schizophrenia Care After Onset of the COVID-19 Pandemic
Source: JAMA Netw Open. 2025 Jan 16;8(1):e2454776. doi: 10.1001/jamanetworkopen.2024.54776 (PMC11739993; doi:10.1001/jamanetworkopen.2024.54776)
Supplement: Supplement 1. — eAppendix 1. Agency Level Diffusion Outcome: Approach and Methodological Details eAppendix 2. Beneficiary Likelihood of Any Telemental Health Visit, Its Association With Race and Ethnicity, and Modification of the Association by Healthcare System Stress: Details on Methods eTable. Hazard Ratios of Time to First Telemental Health Visit in New York State Among 30 990 Medicaid Adults With Schizophrenia eFigure 1. COVID-19 Hospitalization Rates (per 10 000) Reflect Varying Disease Severity, by Date and New York State Catchment Area eFigure 2. CONSORT Diagram eFigure 3. Average Daily Telemental Health Visits Relative to Average In-Person Mental Health Outpatient Visits for New York State Medicaid Beneficiaries With Schizophrenia, Across Agencies eFigure 4. Days to Diffusion Targets eReferences. [file jamanetwopen-e2454776-s001.pdf]

# Supplementary Online Content

Normand SL, Leckman-Westin E, Finnerty M, et al. Race and ethnicity and diffusion of telemedicine in Medicaid for schizophrenia care after onset of the COVID-19 pandemic. *JAMA Netw Open.* 2025;8(1):e2454776. doi:10.1001/jamanetworkopen.2024.54776

**eAppendix 1.** Agency Level Diffusion Outcome: Approach and Methodological Details

**eAppendix 2.** Beneficiary Likelihood of Any Telemental Health Visit, Its Association With Race and Ethnicity, and Modification of the Association by Healthcare System Stress: Details on Methods

**eTable.** Hazard Ratios of Time to First Telemental Health Visit in New York State Among 30,990 Medicaid Adults With Schizophrenia

**eFigure 1**. COVID-19 Hospitalization Rates (per 10,000) Reflect Varying Disease Severity, by Date and New York State Catchment Area

**eFigure 2**. CONSORT Diagram

**eFigure 3**. Average Daily Telemental Health Visits Relative to Average In-Person Mental Health Outpatient Visits for New York State Medicaid Beneficiaries With Schizophrenia, Across Agencies

**eFigure 4**. Days to Diffusion Targets

**eReferences.**

This supplementary material has been provided by the authors to give readers additional information about their work.

© 2025 Normand SL et al. *JAMA Network Open.*

**eAppendix 1.** Agency Level Diffusion Outcome: Approach and Methodological Details

Our primary focus is on the determinants of telemental heath diffusion at the agency level. Diffusion is characterized by **adoption behavior parameters** as depicted in the Figure (saturation levels, within-agency adoption rates (slopes), and origins). Researchers have studied the diffusion of various medical technologies, including computed tomography scans,[1] intensive care units,[2] angioplasty,[3] and robotic surgery.[4] In this paper, we distinguish the effects of agency structure (e.g., hospital-affiliated, free-standing, measures of diversity of Medicaid beneficiary populations served and percent of patients with schizophrenia among them) on the time at which an agency uses a targeted percent of telemental health. The diffusion targets are computed first by calculating the daily cumulative fractions of all mental health visits that are telemental health visits, $C_{it}$. $Let\ Z_{it} = \sum_{j=1}^{t} Y_{ij}\ denote\ the\ cumulative\ number\ of\ telemental\ health\ visits\ by\ day\ t\ for\ agency\ i$ .

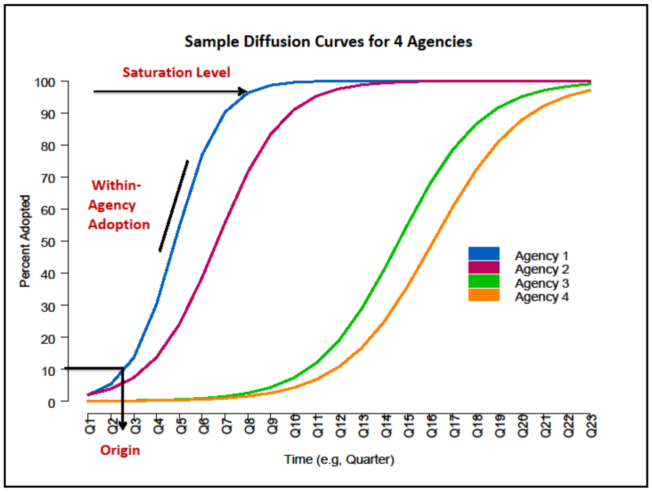


$Let\ m_{it} = \sum_{j=1}^{t} n_{ij}\ denote\ the\ cumulative\ number\ of\ all\ mental\ health\ visits\ by\ day\ t\ for\ agency\ i.$

$C_{it} = \frac{Z_{it}}{m_{it}}\ is\ the\ cumulative\ fraction\ of\ telehealth\ visits\ by\ day\ t\ for\ agency\ i.$

We model the event:

$$a_i = 1\ if\ C_{it} > b\ where\ b\ is\ a\ diffusion\ target\ and\ 0\ otherwise$$

where *b* is selected as 0.10 and 0.50. Referring to the Figure, diffusion is slower in hypothetical Agencies 3 and 4, with time to 10% cumulative adoption achieved in quarter 10 or 11 compared with quarters 2 and 3 for Agencies 1 and 2. All agencies in the Figure reach saturation of 100%, with Agencies 1 and 2 reaching saturation faster than Agencies 3 and 4.

© 2025 Normand SL et al. *JAMA Network Open.*

**eAppendix 2.** Beneficiary Likelihood of Any Telemental Health Visit, Its Association With Race and Ethnicity, and Modification of the Association by Healthcare System Stress: Details on Methods

| Time-Period | New York City | Western + Central NY |
|---|---|---|
| **Period 1**: 3/11/2020 to 4/30/2020 | High stress | Low stress |
| **Period 2**: 7/9/2020 to 8/20/2020 | Low stress | Low stress |
| **Period 3**: 12/24/2020 to 2/4/2021 | Moderate stress | Moderate Stress |

The distribution of minorities differs between New York City and Central/Western New York State, which could confound the association between race and ethnicity and the outcome for this analysis, and the stress of the healthcare system varied according to the severity of the pandemic, which could also confound this association. We selected the New York City and Central/Western catchment areas because of these differences to determine if our overall findings held. The logistic regression model included the main effects of age, SSI, OUD, non-opioid SUD, participation in an opioid treatment program, time-period (as defined in the table), race and ethnicity (with White race as reference), and area (with New York City as reference), and the following terms:

$$Race \times Time - Period + Race \times Central + Central \times Time - Period + Race \times Time - Period \times Central$$

We examined race and ethnicity "odds ratios" within each area and time-period; next, we examined if the "odds ratios" differed between time-periods.

© 2025 Normand SL et al. *JAMA Network Open.*

## Supplemental Tables and Figures

| **eTable.** Hazard Ratios of Time to First Telemental Health Visit in New York State Among 30,990 Medicaid Adults With Schizophrenia. 15% (4,542) of beneficiaries were censored. Estimates adjusted for beneficiary county of residence. | |
|---|---|
| Variable | Hazard Ratio (95% Confidence Interval) |
| Female | 1.07 (1.04, 1.10) |
| 18 – 50 years vs 51 – 64 years | 0.99 (0.96, 1.01) |
| Race and ethnicity (Reference = White) | |
| Asian/Other | 0.93 (0.88, 0.98) |
| Black | 0.90 (0.87, 0.93) |
| Latinx | 0.95 (0.91, 0.99) |
| Unknown | 0.91 (0.87, 0.96) |
| Non-Supplemental security income recipient | 0.94 (0.91, 0.96) |
| Opioid use disorder comorbidity | 0.68 (0.63, 0.73) |
| Participating in an opioid treatment program | 1.41 (1.27, 1.56) |
| Non-opioid substance use disorder comorbidity | 0.55 (0.53, 0.56) |

© 2025 Normand SL et al. *JAMA Network Open.*

**eFigure 1**. COVID-19 Hospitalization Rates (per 10,000) Reflect Varying Disease Severity, by Date and New York State Catchment Area. Arrows indicate 3 different time-periods of healthcare system stress examined.

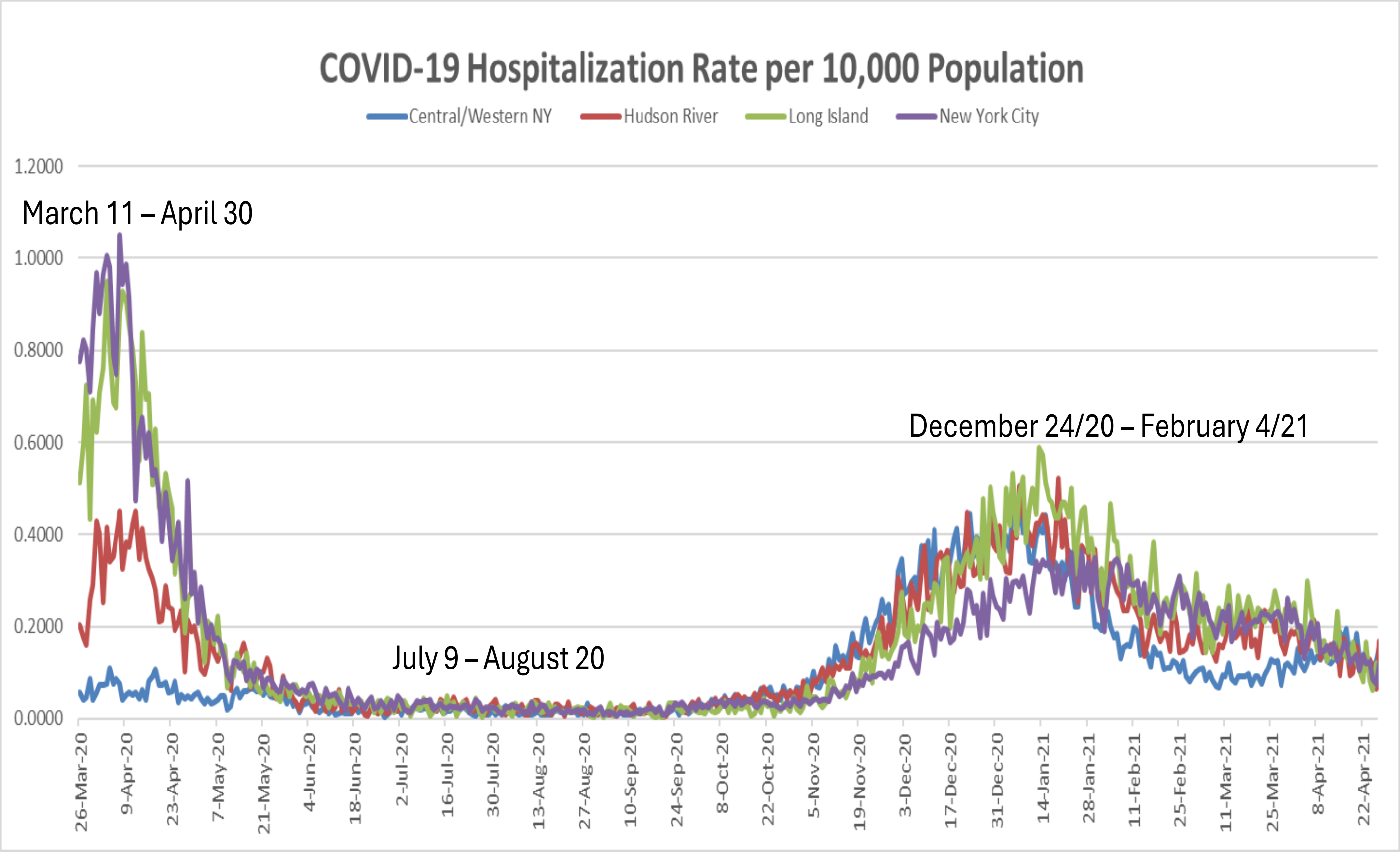


© 2025 Normand SL et al. *JAMA Network Open.*

**eFigure 2.** CONSORT Diagram

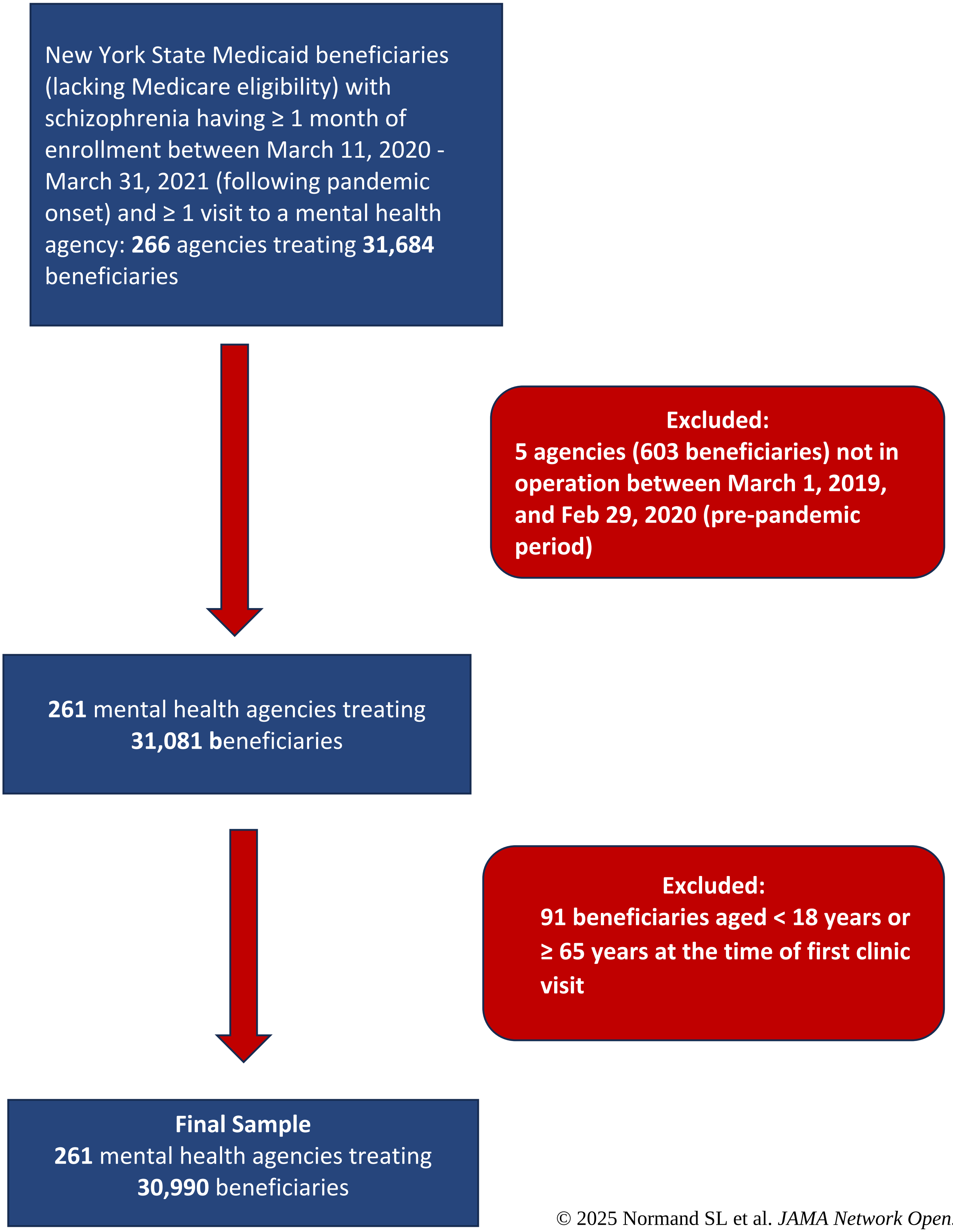


© 2025 Normand SL et al. *JAMA Network Open*.

**eFigure 3**. Average Daily Telemental Health Visits Relative to Average In-Person Mental Health Outpatient Visits for New York State Medicaid Beneficiaries With Schizophrenia, Across Agencies

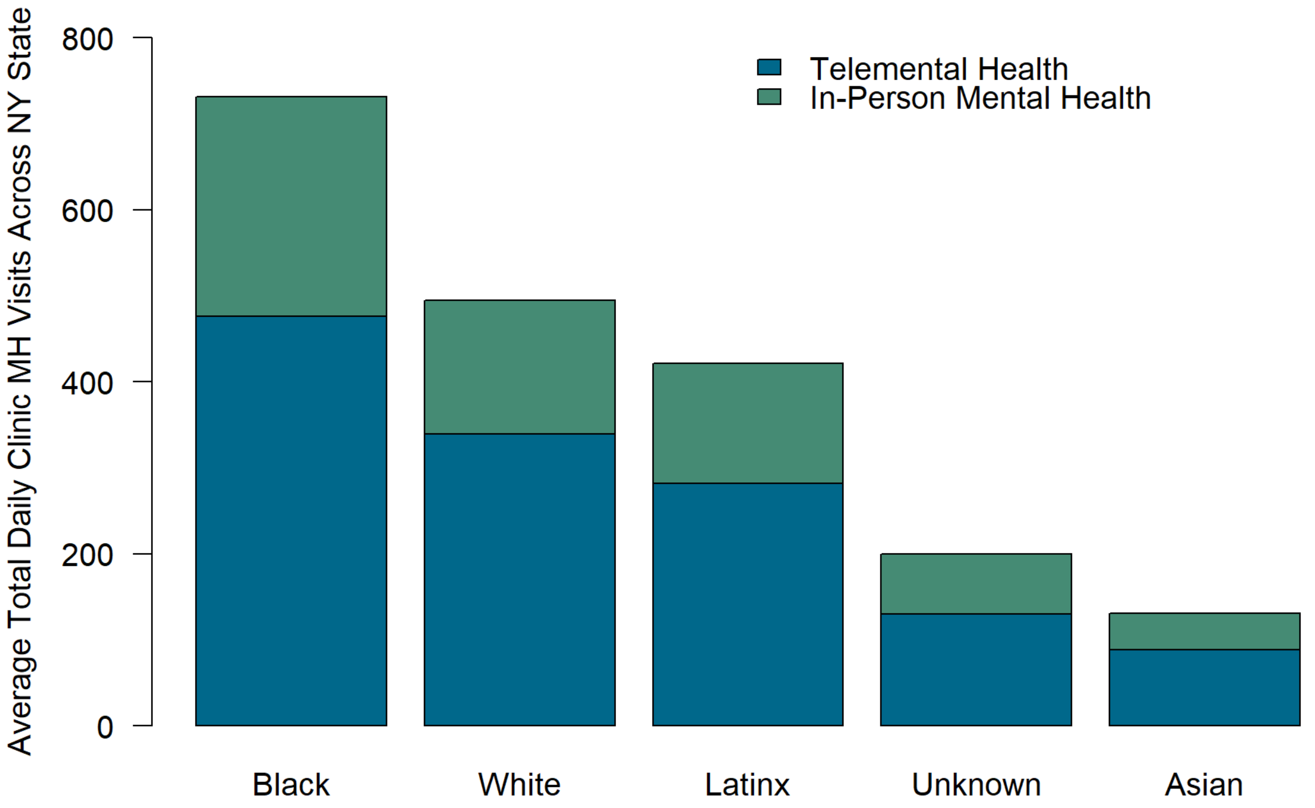


© 2025 Normand SL et al. *JAMA Network Open.*

**eFigure 4**. Days to Diffusion Targets. Vertical lines represent 95% confidence intervals. The numbers below the vertical lines are the number of agencies meeting the target.  Agencies in higher targets appear in the lower targets.

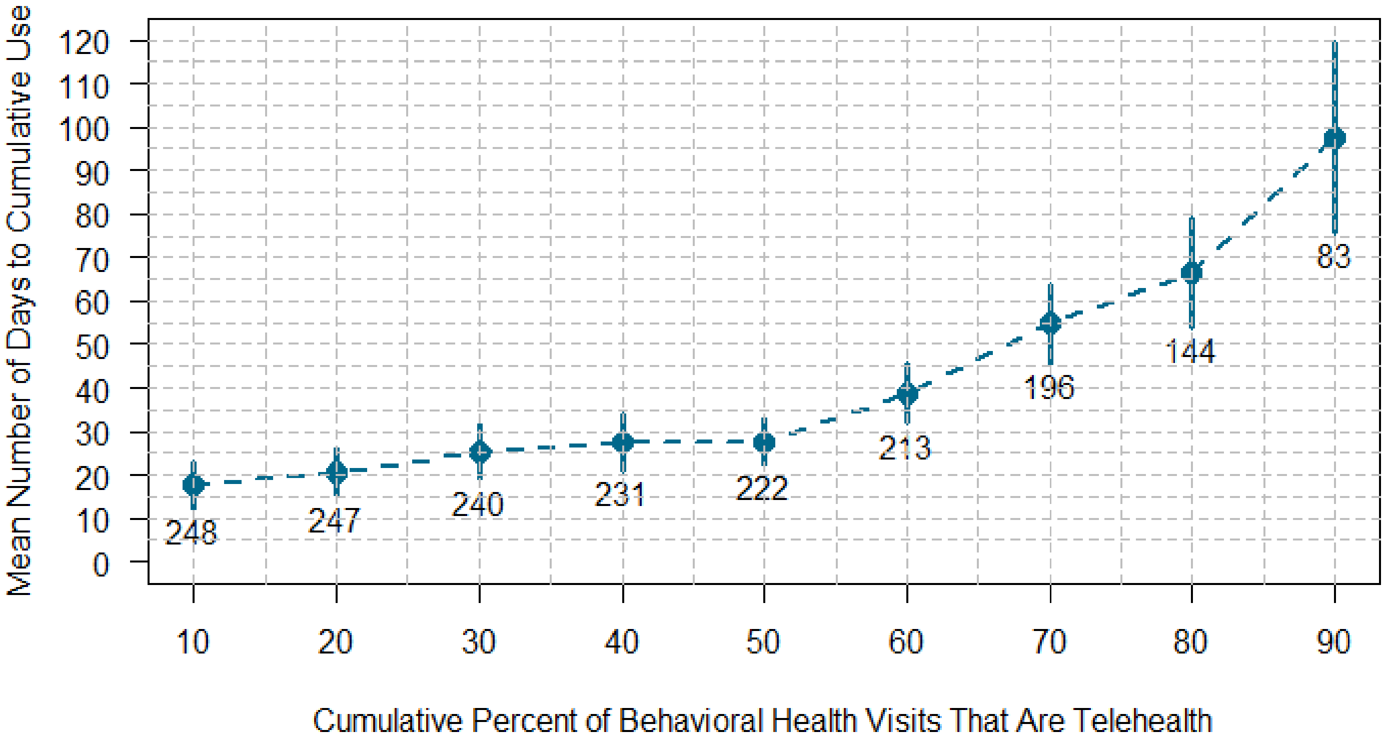


© 2025 Normand SL et al. *JAMA Network Open*.

**eReferences.**

1. Trajtenberg M, Yitzhaki S. The Diffusion of Innovations: A Methodological Reappraisal. *Journal of Business & Economic Statistics*. 1989;7(1):35-47. doi:10.2307/1391836
2. Russell LB. The diffusion of hospital technologies: some econometric evidence. *J Hum Resour*. Fall 1977;12(4):482-502.
3. Cutler DM, Huckman RS. Technological development and medical productivity: the diffusion of angioplasty in New York state. *J Health Econ*. Mar 2003;22(2):187-217. doi:10.1016/s0167-6296(02)00125-x
4. Maynou L, Pearson G, McGuire A, Serra-Sastre V. The diffusion of robotic surgery: Examining technology use in the English NHS. *Health Policy*. Apr 2022;126(4):325-336. doi:10.1016/j.healthpol.2022.02.007

© 2025 Normand SL et al. *JAMA Network Open.*
