# Supplementary material for: Race and Ethnicity and Diffusion of Telemedicine in Medicaid for Schizophrenia Care After Onset of the COVID-19 Pandemic
Source: JAMA Netw Open. 2025 Jan 16;8(1):e2454776. doi: 10.1001/jamanetworkopen.2024.54776 (PMC11739993; doi:10.1001/jamanetworkopen.2024.54776)
Supplement: Supplement 2. — Data Sharing Statement [file jamanetwopen-e2454776-s002.pdf]

Data Sharing Statement

Normand. Race and Ethnicity and Diffusion of Telemedicine in Medicaid for Schizophrenia Care After Onset of the COVID-19 Pandemic. *JAMA Netw Open*. Published January 16, 2025. doi:10.1001/jamanetworkopen.2024.54776

Data
Data available: No

Additional Information
Explanation for why data not available: Data are owned by the Government
